# Acute pancreatitis caused by tigecycline: A case report and literature review

**DOI:** 10.1097/MD.0000000000028245

**Published:** 2021-12-23

**Authors:** Peng-fei Wang, Hong Zou, Ji-hong Zhu, Fang-e Shi

**Affiliations:** Peking University People's Hospital, Beijing, China.

**Keywords:** acute pancreatitis, case report, tigecycline

## Abstract

**Rationale:**

There is evidence that tigecycline has broad-spectrum antibiotic activity against a variety of complicated infections. However, adverse effects are inevitable, including gastrointestinal side effects such as nausea, vomiting, and diarrhea; in 2006, acute pancreatitis was also brought into the side-effect list after postmarketing surveillance. Here, we present a case of tigecycline-induced acute pancreatitis.

**Patient concerns:**

An 87-year-old female patient with urinary tract infection received an intravenous drip of tigecycline for 6 days, after which she developed abdominal distension, vomiting, abdominal pain, and abdominal rigidity.

**Diagnosis:**

The patient was suspected to have tigecycline-induced acute pancreatitis.

**Interventions:**

Tigecycline was discontinued immediately, and the patient received a series of immediate treatments including an indwelling gastric tube for continuous gastrointestinal decompression and inhibition of gastric acid and pancreatic enzyme secretion.

**Outcomes:**

Following initial interventions, we observed that the patient's symptoms improved significantly, and abdominal distension, vomiting, abdominal pain, and abdominal rigidity were slightly relieved. After 5 days of follow-up, blood lipase and amylase levels decreased to normal levels. Unfortunately, the patient developed convulsions during the use of multiple antibiotics after 1 week and then died of septic shock and acute liver failure.

**Lessons:**

Acute pancreatitis caused by tigecycline is rare. However, in the application of antibiotics, the possibility of adverse effects must be considered, and antibiotics should be used reasonably. If the patient has relevant symptoms, it is necessary to stop using tigecycline immediately, carry out symptomatic treatment, and change to other types of antibiotics for antibacterial treatment.

## 1 Introduction

There is evidence that tigecycline has broad-spectrum antibiotic activity against a variety of complicated infections. However, adverse effects are inevitable, including gastrointestinal side effects such as nausea, vomiting, and diarrhea; in 2006, acute pancreatitis was also brought into the side-effect list after postmarketing surveillance. The incidence rate of tigecycline-induced acute pancreatitis was relatively low. This suggests that we should have a more comprehensive understanding of the adverse reactions of tigecycline and other drugs. Here, we present a case of tigecycline-induced AP and review the literature on tigecycline-induced acute pancreatitis.

## 2 Case presentation

An 87-year-old woman was repeatedly hospitalized for urinary tract infections in the past 4 months. She was allergic to penicillin and sulfa drugs. During treatment, the effect of multiple-adjusted antibacterial therapy strategies was poor. Urine bacterial culture results showed carbapenem-resistant Klebsiella pneumonia 2 months previously, and the results of the drug sensitivity test showed that it was sensitive to tigecycline and sulbactam sodium for injection, and the patient was treated with sulbactam sodium for injection and tigecycline, her symptoms improved, and she was discharged from the hospital. However, the patient reappeared turbid urine and many flocs 4 days prior, and was administered nitrofurantoin, sulbactam sodium for injection, and minocycline in an outside hospital; the urine was still turbid, leaving a small number of flocs and was admitted to our emergency department.

On presentation, the patient was admitted to the hospital with a state of lethargy, with stable vital signs, a soft abdomen without tenderness, and slight pitting edema in both lower limbs. Tigecycline was administered intravenously at 100 mg for the first dose and then administered at 50 mg every 12 h combined with etimicin 300 mg every 24 hours because of persistent urinary tract infections. Six days after intravenous infusion, the patient suddenly presented with abdominal distension, vomiting, abdominal pain, and abdominal rigidity that refused to be pressed.

**Figure 1:**
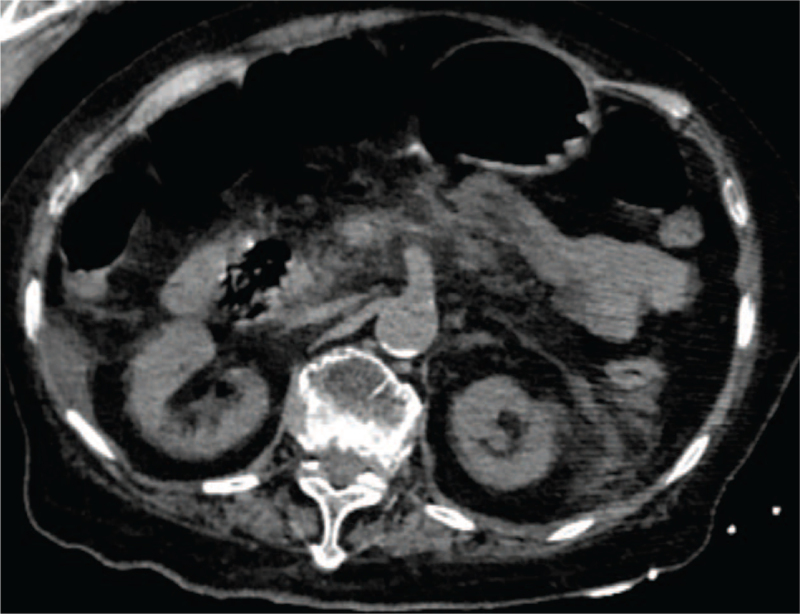
Abdominal and pelvic computed tomography (CT) revealed that an irregular pancreatic shape in the abdominal cavity, with the normal pancreatic duct. Blurred fat space around the pancreas, and there was little effusion in the abdominal and pelvic cavity.

Urgent laboratory analyses indicated the following: white blood cell count, 31.25 × 10^9^/L; procalcitonin, 1 ng/mL; C-reactive protein is 67.9 mg/L, blood amylase, 939 U/L (reference value 28–100 U/L), lipase 2552.6 U/L (reference value 13–60 U/L), urine amylase, 2067 U/L (reference value <460 U/L), IgG4 negative. Abdominal and pelvic computed tomography (Fig. 1) revealed acute pancreatitis, an irregular pancreatic shape in the abdominal cavity, with a normal pancreatic duct and blurred fat space around the pancreas, with little effusion in the abdominal and pelvic cavity. Gastroenterology specialists confirmed the diagnosis of acute pancreatitis. Common causes of pancreatitis include biliary tract disease, alcohol intake, hypertriglyceridemia, hypercalcemia, trauma and surgery, and immune factors. Finally, these findings were considered to be related to drug-induced pancreatitis, and it was believed that acute pancreatitis caused by tigecycline was established. Tigecycline was discontinued immediately on the 6th day following exposure, after a series of treatments including an indwelling gastric tube for continuous gastrointestinal decompression, inhibition of gastric acid, and pancreatic enzyme secretion for approximately 2 days, and abdominal distension and other symptoms were slightly relieved. Her blood lipase level decreased to 607 U/L, and the amylase level decreased to 824 U/L. After 5 days of follow-up, the patient's symptoms improved significantly, blood lipase and amylase levels decreased to normal levels, and the patient felt well and asked to be discharged. Unfortunately, after 1 week, the patient developed convulsions during the use of multiple antibiotics, and then died of septic shock and acute liver failure.

## 3 Discussion

Tigecycline was the only drug in the glycylcycline class of antibacterial agents for clinical use. It was approved by the Food and Drug Administration in 2005 and was approved by China in 2011. It has broad-spectrum antibacterial activity against gram-positive bacteria, gram-negative bacteria, anaerobic bacteria, methicillin-resistant *Staphylococcus aureus*, penicillin-resistant *Streptococcus pneumoniae*, vancomycin-resistant *Enterococcus*, and resistant *Acinetobacter baumannii*.^[1]^ It has been approved for use in complicated skin and soft tissue infections, complicated intra-abdominal infections, and community-acquired pneumonia.^[2,3]^ According to relevant data, the incidence of pancreatitis caused by tigecycline is approximately 0.1% to 1%.^[4]^ In a phase III clinical trial of tigecycline, it was found that the incidence of tigecycline increased serum amylase was 3.1%, and the incidence of abdominal pain was 4.5%,^[5–8]^ and the main clinical symptoms were nausea, vomiting, abdominal pain, abdominal distension, and diarrhea.

The incidence of acute pancreatitis is increasing worldwide,^[9]^ gallstones and alcohol intake are the main causes of acute pancreatitis.^[10]^ Drug-induced pancreatitis is a special type of pancreatitis caused by drugs. Since the 1950s, there have been reports of drug-induced pancreatitis because of the lack of specific laboratory test indicators and imaging features, and the variety of drugs that can cause acute pancreatitis, it is difficult to distinguish acute pancreatitis from other causes. The latest report points out that the total incidence of drug-induced pancreatitis is 0.3% to 5.3%.^[11]^ Drugs related to acute pancreatitis include tetracycline, macrolide, isoniazid, and metronidazole.^[12,13]^ Relevant literature data in recent years have shown that reports of tigecycline-induced acute pancreatitis have increased significantly.^[4,14,15]^

The mechanism by which tigecycline causes pancreatitis is not clear, but it is a structural derivative of minocycline. Its chemical structure, pharmacokinetic characteristics, and adverse reactions were similar to those of tetracycline drugs. Therefore, there are 3 different mechanism hypotheses^[16,17]^: toxic metabolites, hypertriglyceridemia, and high bile concentration.

Search the database with “tigecycline and pancreatitis” as keywords, and the search time range is from 2005 to 2021. A total of 37 articles were searched, and irrelevant articles were removed, and 11 cases from 10 case reports were obtained. At the same time, the patient's gender, age, infection type, clinical manifestations of acute pancreatitis, pathogenic bacteria, blood amylase, and lipase were counted from the literature. There were 7 male and 4 female patients, ranging in age from 9 to 69 years. Among the infection types, there were 5 cases of tissue infection, 2 cases of blood infection, 1 case of lung infection, 2 cases of osteomyelitis, and 1 case of donor infection. The results are summarized in Table 1.

**Table 1:** Clinical manifestations, blood amylase, lipase test results, and clinical manifestations of patients with tigecycline-induced pancreatitis.

Author	Age	Gender	Type of infection	Onset symptoms	Pathogenic bacteria	Blood amylase (UI/L)	Lipase (UI/L)
Lin et al^[18]^	48	Female	donor-derived infection after kidney transplantation	Recurrent fever and abdominal distension	*Acinetobcter baumannii*	424	156
Akhter et al^[14]^	61	Male	leg infection from immunosuppressive therapy	Nausea, vomiting, and worsening epigastric abdominal pain	Nontuberculous, *Mycoplasma*, chelonae	21000	1835
Hung et al^[12]^	69	Female	complicated skin-structure infection	Persistent and worsening nausea and vomiting, severe abdominal pain	Coagulase-negative *Staphylococcus epidermidis*	926	749
Marot et al^[19]^	64	Male	Toe infection	Nausea, epigastric pain	*Staphylococcus aureus*, susceptible to oxacillin, and resistant to clindamycine, and penicillin	–	936
Marot et al^[19]^	58	Male	Osteonecrosis and infection of the phalanx	Nausea, vomiting and loss of appetite	Coagulase-negative Staphylococci – *Staphyloccus scleiferi*, methicillin-resistant, and *Staphylococcus lugdunensis*, methicillin-sensitive	552	1660
Lipshitz et al^[20]^	64	Female	Prosthetic hip infection	Severe epigastric pain associated with nausea and vomiting	–	806	1406
Hemphill and Jones^[21]^	22	Male	Cystic fibrosis, lung infection	Epigastric tenderness as well as mild nausea	*Mycobacterium chelonae*	381	268
Bernas Albeniz et al^[22]^	68	Female	Septicemia	Abdominal pain	–	406	1094
Gilson et al^[4]^	35	Male	Chronic osteitis and sinus tract discharge	Nausea and vomiting	*Enterobacter cloacae* with broad-spectrum beta-lactamase	–	–
Prot-labarthe et al^[23]^	9	Male	Septicemia	Abdominal pain, recurrent vomiting	*Enterobacter cloacae*	–	603
Mascarello et al^[24]^	–	Male	Chronic osteomyelitis	Nausea, vomiting and acute severe upper abdominal pain	Methicillin-resistant *Staphylococcus aureus*, multidrug-resistant *Pseudomonas aeruginosa* and *Acinetobacter baumannii*	312	382

## 4 Conclusion

Acute pancreatitis caused by tigecycline is a relatively rare phenomenon. Currently, there is no more sensitive identification method to prevent this adverse reaction during tigecycline treatment. In the process of clinical application, once the patient shows persistent and severe nausea, vomiting, and abdominal pain, it is necessary to rule out the possibility of pancreatitis as soon as possible. In the future, more attention should be paid to the mechanism of tigecycline-induced acute pancreatitis.

## Author contributions

**Investigation:** Peng-fei Wang, Hong Zou.

**Methodology:** Peng-fei Wang.

**Project administration:** Ji-hong Zhu.

**Supervision:** Ji-hong Zhu, Fang-e Shi.

**Writing – original draft:** Peng-fei Wang, Fang-e Shi.

**Writing – review & editing:** Ji-hong Zhu, Fang-e Shi.
